# Light and the circadian clock mediate time-specific changes in sensitivity to UV-B stress under light/dark cycles

**DOI:** 10.1093/jxb/eru339

**Published:** 2014-08-21

**Authors:** Tomomi Takeuchi, Linsey Newton, Alyssa Burkhardt, Saundra Mason, Eva M. Farré

**Affiliations:** Department of Plant Biology, Michigan State University, East Lansing, MI 48824, USA

**Keywords:** Circadian clock, gating, light, signalling, transcription, UV-B.

## Abstract

In *Arabidopsis*, the circadian clock regulates UV-B-mediated changes in gene expression. Here it is shown that circadian clock components are able to inhibit UV-B-induced gene expression in a gene-by-gene-specific manner and act downstream of the initial UV-B sensing by COP1 (CONSTITUTIVE PHOTOMORPHOGENIC 1) and UVR8 (UV RESISTANCE LOCUS 8). For example, the UV-B induction of *ELIP1* (*EARLY LIGHT INDUCIBLE PROTEIN 1*) and *PRR9* (*PSEUDO-RESPONSE REGULATOR 9*) is directly regulated by LUX (LUX ARRYTHMO), ELF4 (EARLY FLOWERING 4), and ELF3. Moreover, time-dependent changes in plant sensitivity to UV-B damage were observed. Wild-type *Arabidopsis* plants, but not circadian clock mutants, were more sensitive to UV-B treatment during the night periods than during the light periods under diel cycles. Experiments performed under short cycles of 6h light and 6h darkness showed that the increased stress sensitivity of plants to UV-B in the dark only occurred during the subjective night and not during the subjective day in wild-type seedlings. In contrast, the stress sensitivity of *Arabidopsis* mutants with a compromised circadian clock was still influenced by the light condition during the subjective day. Taken together, the results show that the clock and light modulate plant sensitivity to UV-B stress at different times of the day.

## Summary

The circadian clock and light influence the time dependence of the UV-B stress response. Circadian clock components regulate UV-B-mediated expression in a gene-by-gene-specific manner in *Arabidopsis*.

## Introduction

Circadian clocks are biological molecular oscillators that generate rhythms of ~24h. They rhythmically coordinate many key molecular and physiological processes to the daily and seasonal changes in the environment, and they are ubiquitously present in most living organisms exposed to cycles of day and night (Bell-Pedersen *et al.*, 2005). In eukaryotic organisms including *Arabidopsis thaliana*, the circadian clock constitutes a complex regulatory network formed by multiple interlocked transcriptional and translational feedback loops (Nagel and Kay, 2012). For example, in *Arabidopsis*, the morning-expressed Myb transcription factors CIRCADIAN CLOCK ASSOCIATED (CCA1) and LATE ELONGATED HYPOCOTYL (LHY) activate the transcription of *PSEUDO RESPONSE REGULATOR 7* (*PRR7*) and *9* (*PRR9*) in the morning (Farre *et al.*, 2005). In turn, the pseudo-response regulators PRR7, PRR9, PRR5, and TOC1/PRR1 (*TIMING OF CHLOROPHYLL A/B BINDING PROTEN*) proteins inhibit the transcription of *CCA1/LHY* during the day and throughout the evening (Farre and Kay, 2007; Nakamichi *et al.*, 2010; Huang *et al.*, 2012). CCA1 and LHY repress the expression of the evening-expressed genes *TOC1*, *EARLY FLOWERING 3* (*ELF3*), and *ELF4*, and the transcription factor *LUX ARRYTHMO* (*LUX*) (Nakamichi, 2011). A protein complex composed of ELF3, ELF4, and LUX (evening complex; EC) was found to regulate the expression of *PRR9* directly (Dixon *et al.*, 2011; Helfer *et al.*, 2011; Chow *et al.*, 2012; Herrero *et al.*, 2012).

The circadian clock regulates ~30% of the genes in angiosperm genomes (Covington *et al.*, 2008; Michael *et al.*, 2008; Khan *et al.*, 2010; Filichkin *et al.*, 2011), and the integration of circadian, environmental, and internal signals sets the timing of gene expression such that 60–100% of the genome in photosynthetic organisms cycles under diurnal conditions (Michael *et al.*, 2008; Monnier *et al.*, 2010; Filichkin *et al.*, 2011). Moreover, recent studies show that many of these genes are directly regulated by circadian clock components, providing a mechanism for the influence of the clock on plant growth, development, and stress responses (Huang *et al.*, 2012; Nakamichi *et al.*, 2012; Liu *et al.*, 2013). One of the roles of the clock is to modulate the response to external stimuli at different times of day, a phenomenon defined as ‘gating’. In *Arabidopsis*, the clock gates not only visible light signalling responses but also low-intensity UV-B-mediated changes in gene expression (McWatters *et al.*, 2000; Feher *et al.*, 2011). Thus the magnitude of the change in RNA levels after UV-B exposure depends on the time of day of the treatment (Feher *et al.*, 2011).

UV-B light (280–315nm) is a natural component of sunlight, and, due to its short wavelength, it has the highest energy of the sunlight spectrum at the Earth’s surface (Jansen *et al.*, 1998). While high-intensity UV-B light causes damage to DNA, protein, and other macromolecules (Jansen *et al.*, 1998), low fluence UV-B light promotes photomorphogenesis, and induces the transcription of genes involved in flavonoid synthesis (Jenkins, 2009; Li *et al.*, 2013). The UV RESISTANCE LOCUS 8 (UVR8) was recently elucidated as the photoreceptor of UV-B irradiation in plants (Rizzini *et al.*, 2011). In the absence of UV-B light, UVR8 primarily exists as a homodimer *in vivo* and *in vitro*, and it monomerizes rapidly following UV-B photoreception (Rizzini *et al.*, 2011; Christie *et al.*, 2012; Wu *et al.*, 2012). The monomeric UVR8 then accumulates in the nucleus and interacts with COP1 (CONSTITUTIVELY PHOTOMORPHOGENIC 1) protein to regulate UV-B-dependent responses (Kaiserli and Jenkins, 2007; Cloix *et al.*, 2012).

Many of the responses to UV-B involve the regulation of gene expression. Among the genes thus regulated is one that encodes the transcription factor HY5 (ELONGATED HYPOCOTYL 5), which also accumulates in the nucleus following UV-B irradiation (Oravecz *et al.*, 2006). HY5 and its homologue, HYH (HY5 homologue), extensively mediate UV-B-dependent gene expression and regulate the UV-B-induced photomorphogenic pathway (Ulm *et al.*, 2004; Brown *et al.*, 2005; Oravecz *et al.*, 2006). However, the UV-B-dependent induction of clock genes such as *CCA1* and *PRR9* is independent of HY5 and HYH (Feher *et al.*, 2011). Moreover, despite the role of HY5 and HYH as the main regulators of UV-B-mediated gene expression in *Arabidopsis*, the circadian gating of UV-B-induced gene expression was shown to occur in a HY5- and HYH-independent manner (Feher *et al.*, 2011). In the same study, it was shown that lines with disturbed circadian rhythms displayed non-cycling constitutive gene induction by UV-B, although the mechanism by which the circadian clock regulates UV-B signalling is not understood (Feher *et al.*, 2011).

It is expected that adaptation to changes in UV-B irradiation during the day is essential to the survival of the plants in nature. However, the role of circadian gating of UV-B signalling in the adaptation of plants to UV-B stress remains unclear. For example, no difference in UV-B stress sensitivity had been observed in plants irradiated at different times of the circadian cycle or between the wild type and circadian mutant plants with constitutively high UV-B-mediated gene induction (Feher *et al.*, 2011). In this study, the aim was to investigate the role of circadian clock components in the regulation of UV-B-mediated gene expression and the role of the clock in changes in UV-B stress sensitivity throughout the day.

## Materials and methods

### Plant material

Lines PRR7ox (*35S::HAPRR7* #54) (Farre and Kay, 2007), CCA1ox (CCA1ox #34) (Wang and Tobin, 1998), *cca1lhy* (*cca1-11 lhy-21*, CS9380) (Dong *et al.*, 2011), *lux-4* (Hazen *et al.*, 2005b), *elf3-1* (CS3787) (Hicks *et al.*, 2001), *elf3-8* (CS3794) (Hicks *et al.*, 2001), *cop1-4* (McNellis *et al.*, 1994), *prr5prr7prr9* (Liu *et al.*, 2013), *ELF4::HA-ELF4 elf4-2* (Nusinow *et al.*, 2011), *LUX::LUX-GFP lux-4* (Helfer *et al.*, 2011), *cop1elf3* (*cop1-4 elf3-8)* (Yu *et al.*, 2008), *CCA1pro::LUC* (Pruneda-Paz *et al.*, 2009), *LHYpro::LUC* (Pruneda-Paz *et al.*, 2009), *PRR9pro::LUC* (Para *et al.*, 2007), and *CHSpro::LUC* (Brown *et al.*, 2005) were described previously. The line *cop1-4 lux-4* was generated by crossing. The mutant *elf4-300* was identified in a mutant screen described previously (Hazen *et al.*, 2005a); it contains the mutation G78A leading to a premature stop codon (W26*). All the lines with the exception of *cca1lhy* (Ws) and *CHSpro::LUC* (Ler) are in the Col-0 background.

### UV-B light treatments

An XX-15M model UV-B lamp (peak at 302nm; UVP, Upland, CA, USA) was used for all UV-B treatments. The light was filtered through coloured glass alternative longpass filters from Newport Stabilife Technology (65CGA-345 or 65CGA305) with a cut-on wavelength, which denotes the wavelength at which the transmission increases to 50% throughput in a longpass filter, of 345nm (control) or 305nm (UV-B) unless otherwise stated. The UV-B output of the lamp (280–320nm) was monitored with a PS-200 spectroradiometer (Apogee Instruments, Logan, UT, USA). The spectra of the irradiances used are shown in Supplementary Fig. S1 available atr *JXB* online. The full lamp spectrum is available from UVP.

### Analysis of UV-B-induced gene expression by quantitative real-time PCR

Seedlings were grown on Murashige and Skoog (MS) medium (Murashige and Skoog, 1962) for 15 d under light/dark (12h light, 12h dark) conditions before being transferred to constant light (70 μmol m^–2^ s^–1^, 22 °C). Plants were treated with UV-B light for 10min at the respective time points with filters that have a cut-on wavelength of 345nm (control) (0.8 μW cm^–2^/0.02 μmol m^–2^ s^–1^ UV-B) or 305nm (UV-B) (110 μW cm^–2^/3 μmol m^–2^ s^–1^ UV-B) in the presence of 70 μmol m^–2^ s^–1^ white light. After this treatment, they were transferred to white light for 1h 20min and then snap-frozen in liquid nitrogen. RNA was extracted using the EZNA Plant RNA extraction kit (Omega, Norcross, GA, USA). For reverse transcriptase-mediated PCR, 1 μg of total RNA was used with the iScript cDNA synthesis kit (Bio-Rad) according to the manufacturer’s protocol. The resulting cDNA was diluted five times with water, and 1.5 μl of this dilution were used for real-time quantitative PCR using SYBR-Green Master Mix (Applied Biosystems, Warrington, UK) and an Eppendorf single-colour real-time PCR detection system (Master Cycle Realplex^2^). Quantification was carried out by PCR baseline subtracted curve fit with the RealPlex software. Two technical replicates for each of three biological replicates per line/treatment were analysed. The *IPP2* (AT3G02780) gene, which was not induced by UV-B and is not circadian regulated, was used as a normalization control. The primers used are described in Supplementary Table S1 at *JXB* online.

### Chromatin immunoprecipitation

Chromatin immunoprecipitation using *ELF4::HA-ELF4 elf4-2* (Nusinow *et al.*, 2011) and *LUX::LUX-GFP lux-4* (Helfer *et al.*, 2011) was performed as described previously (Liu *et al.*, 2013). Fifteen-day-old *Arabidopsis* seedlings growing on MS medium with 2% sucrose were harvested at Zeitgeber time 12 (ZT12). ZT is defined as hours after the last dark to light transition. For the UV-B-treated samples, seedlings were transferred to 110 μW cm^–2^/3 μmol m^–2^ s^–1^ UV-B using the 305nm longpass filter for 10min, 40min prior to harvesting. Immunoprecipitation was performed with Dynabeads ProteinG (Invitrogen Dynal AS, Oslo, Norway). Beads were pre-treated with anti-HA high-affinity rat IgG monoclonal antibody (clone 3F10, Roche, Basel, Switzerland, 10 μg per 50 μl of beads) or rabbit anti-green fluorescent protein (GFP) polyclonal antibody (Ab290, Abcam, Cambridge, MA, USA; 4 μg per 50 μl of beads). Quantification of immunoprecipitated DNA was carried out by quantitative PCR using the primers listed in Supplementary Table S1 at *JXB* online.

### UV-B stress tolerance assays

Seeds were plated on MS medium without sucrose ~1cm apart. For the UV-B treatment, 10-day-old seedlings were treated with UV-B using the 305nm longpass filter for 10min at the indicated times (110 μW cm^–2^/3 μmol m^–2^ s^–1^ UV-B). After 3h, the seedlings were irradiated with higher intensity UV-B light (293 μW cm^–2^/7.7 μmol m^–2^ s^–1^ UV-B) for 3h. The control seedlings were treated in the same manner but using the 345nm longpass filter at 0.8 μW cm^–2^/0.02 μmol m^–2^ s^–1^ UV-B for the short treatment and 6.3 μW cm^–2^/0.16 μmol m^–2^ s^–1^ UV-B for the long treatment. Seedlings were transferred to conditions of 12h light/12h darkness after the treatments and their weight was analysed 20 d after treatment in pools of 3–5 plants.

### Bioluminescence analysis of UV-B-induced gene expression

Seedlings were grown on MS medium with 2% sucrose for 7–8 d under light/dark (12h light, 12h dark) conditions (70 μmol m^–2^ s^–1^, 22 °C). For experiments under constant light and T-cycles, seedlings were transferred to a 96-well opaque white plate containing solid MS medium with 2% sucrose and each seedling was treated with 30 μl of 5mM luciferin in 0.01% Silwet-77 one day prior to the start of the analysis. Half the plate was UV-B treated using the 305nm longpass filter for 5min to 1h depending on the experiment (110 μW cm^–2^/3 μmol m^–2^ s^–1^ UV-B). The other half of the plate was placed under the UV-B lamp but covered with the 345nm longpass filter and served as control (0.8 μW cm^–2^/0.02 μmol m^–2^ s^–1^ UV-B). Bioluminescence was monitored before and for 3h after the UV-B treatment using a Centro SX3 luminometer (Berthold, Bad Wildbad, Germany). For experiments under constant darkness, seedlings were treated with 5mM luciferin in 0.01% Silwet-77 in the darkness 1 d prior to analysis. Luminescence was monitored using an Andor iKon-M DU-934N-BV camera. Seedlings were treated for 10min with UV-B using the 305nm longpass filter (110 μW cm^–2^/3 μmol m^–2^ s^–1^ UV-B). Seedlings covered with the 345nm longpass filter served as control (0.8 μW cm^–2^/0.02 μmol m^–2^ s^–1^ UV-B). Luminescence was normalized to the corresponding pre-treatment value and the pre-treatment luminescence of their respective controls as reported for similar experiments (Covington and Harmer, 2007).

## Results

### Circadian clock mutants have a disturbed rhythm of UV-B-induced gene expression

**Fig. 1.**
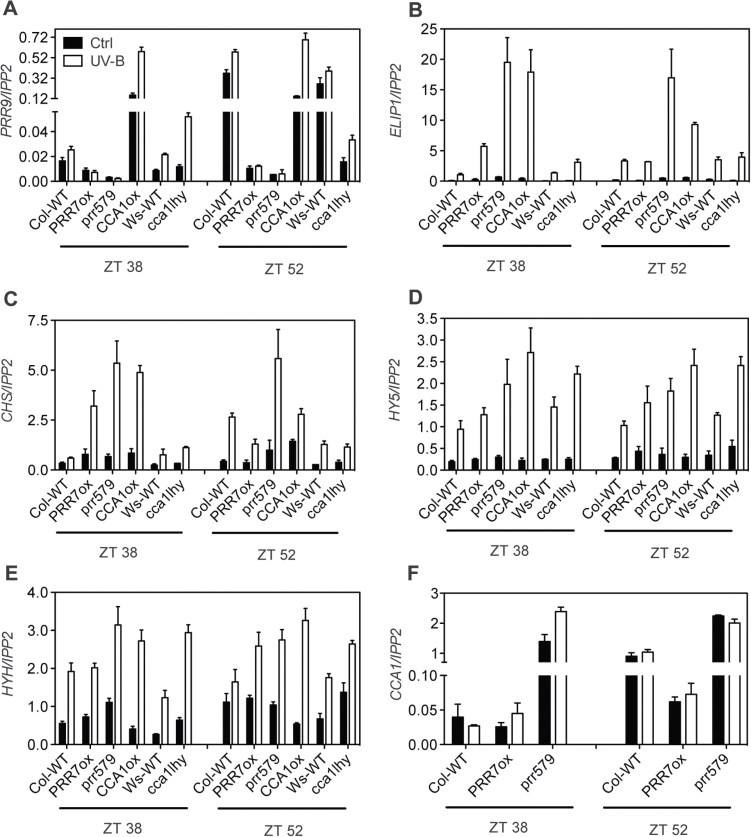
Clock mutants with disturbed circadian rhythms show changes in UV-B-induced gene expression. Two-week-old seedlings were treated with UV-B for 10min at the indicated times under constant light conditions using the 345nm (Ctrl) or the 305nm (UV-B) longpass filter. Samples were harvested 1.5h after the start of the treatment. Values represent the averages and standard errors of three biological replicates. The expression levels of each gene were analysed by RT-qPCR and normalized to *IPP2*.

About 54% of UV-B-induced genes cycle under constant light conditions, indicating that they are circadian regulated (Supplementary Fig. S2A at *JXB* online). The expression of most of these genes peaks during the second half of the subjective night under constant light and during the first half of the day under diel conditions (Supplementary Fig. S2B, C). Therefore, the role of circadian clock components in the regulation of UV-B signalling was investigated. First the UV-B-induced gene expression under constant light conditions was tested in lines that have a severely compromised circadian clock, a PRR7 overexpressor (PRR7ox) (Farre and Kay, 2007), the *prr5 prr7 prr9* triple mutant (*prr579*), as well as the *cca1 lhy* double mutant (Dong *et al.*, 2011) and the CCA1ox line (Fig. 1). It had been previously shown that the arrhythmic *elf3-4* and the CCA1 overexpressor (CCA1ox) display no circadian-regulated gating of UV-B induction of gene expression, such that UV-B light is able to induce gene expression at all times (Feher *et al.*, 2011). The arrhythmic *prr579* triple mutant showed a degree of misregulation in the UV-B response similar to the CCA1ox line (Fig. 1). While an attenuation of UV-B-induced *PRR9*, *CHS* (*CHALCONE SYNTHASE*), and *ELIP1* (*EARLY LIGHT INDUCIBLE PROTEIN 1*) gene expression occurred during the subjective night in wild-type seedlings (ZT 38), the *prr579* triple mutant and CCA1ox showed a constitutively higher expression of these genes at both ZT38 and ZT52 than the wild type, indicating that the circadian gating of UV-B signalling was diminished in these mutants. These results suggest that the PRRs and CCA1 inhibit and promote UV-B-mediated gene expression, respectively. However, most of the genes tested were still induced by UV-B light in the *PRR7ox* and *cca1lhy* plants (Fig. 1). No induction of *CCA1* RNA content after UV-B treatment was observed in wild-type seedlings, despite having been previously reported (Feher *et al.*, 2011) (Fig. 1F). Similar results were obtained using *CCA1pro::LUC* reporter lines, although *PRR9pro::LUC* and *CHSpro::LUC* expression lines displayed UV-B inducibility under the present experimental conditions (Supplementary Fig. S3).

**Fig. 2.**
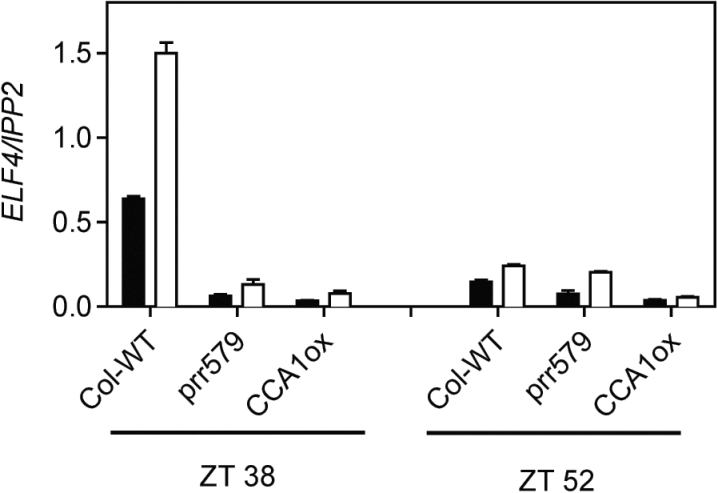
The expression of *ELF4* in CCA1ox and *prr579* seedlings. Two-week-old seedlings were treated with UV-B for 10min at the indicated times under constant light conditions using the 345nm (control, black bars) or the 305nm (UV-B, white bars) longpass filter. Samples were harvested 1.5h after the start of the treatment. Values represent the averages and standard errors of three biological replicates. The expression levels of each gene were analysed by RT-qPCR and normalized to *IPP2*.

Interestingly, the overexpression of *PRR7* not only inhibited *PRR9* transcription under constant light, as had been previously shown (Liu *et al.*, 2013), but also completely blocked the increase in *PRR9* RNA levels after UV-B treatment (Fig. 1A). Since both CCA1ox and *prr579* plants have constitutively high *CCA1* RNA levels (Wang and Tobin, 1998; Nakamichi *et al.*, 2005) (Fig. 1F), which led to the repression of *ELF4* transcription under visible light (Kikis *et al.*, 2005; Li *et al.*, 2011), their effect on *ELF4* expression under UV-B was investigated. A strong inhibition of UV-B mediated *ELF4* induction was observed in both CCA1ox and *prr579* plants (Fig. 2). The expression of *LUX* and *ELF3* was not as strongly affected in these lines (Supplementary Fig. S4 at *JXB* online), although *LUX* is also regulated by CCA1 (Hazen *et al.*, 2005b). Since *PRR9* and *ELF4* are direct targets of PRR7 and CCA1, respectively (Li *et al.*, 2011; Liu *et al.*, 2013), these findings indicate that circadian clock components are able to repress UV-B-mediated transcriptional activation in a gene-by-gene-specific manner. This mechanism explains the apparent absence of a general UV-B gating mechanism (Feher *et al.*, 2011). Thus the circadian clock is able to block UV-B-mediated *ELF4* induction in the morning and allows it at night, but the reverse is true for *PRR9* expression.

**Fig. 3.**
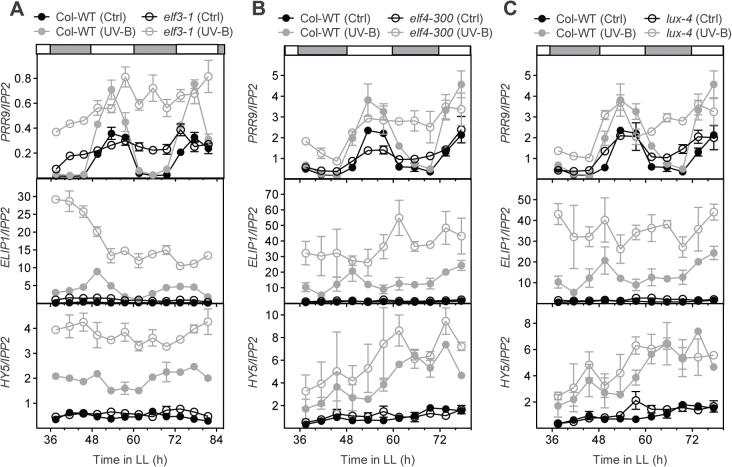
Evening complex mutants show a constitutive response to UV-B irradiation. Expression levels under constant light conditions in (A) *elf3-1*, (B) *elf4-300*, and (C) *lux-4*. Two-week-old seedlings were treated with UV-B for 10min at the indicated times using the 345nm (Ctrl) or the 305nm (UV-B) longpass filter. Samples were harvested 1.5h after the start of the treatment. Values represent the averages and standard errors of three biological replicates. The expression levels of each gene were analysed by RT-qPCR and normalized to *IPP2*.

The EC formed by ELF3, ELF4, and LUX represses the expression of several clock-regulated genes including *PRR9* (Dixon *et al.*, 2011; Nusinow *et al.*, 2011; Helfer *et al.*, 2011; Chow *et al.*, 2012; Herrero *et al.*, 2012). The mutants of *ELF3*, *ELF4*, or *LUX* share similar phenotypes, such as an arrhythmic circadian oscillator in constant light, early flowering, and elongated hypocotyls under diel cycles (Doyle *et al.*, 2002; Hazen *et al.*, 2005b; Nusinow *et al.*, 2011). Thus, given the loss of gating observed in *elf3-4* (Feher *et al.*, 2011), it was tested whether ELF4 and LUX also play a role in the attenuation of UV-B signals during subjective night. As previously reported for *elf3-4*, UV-B-induced gene expression remained constitutively high in *elf3-1*, independent of the time at which the UV-B pulse was given (Fig. 3A; Supplementary S5A at *JXB* online). In both *elf4-300* and *lux-4* mutants, the UV-B-induced expression of *PRR9*, *CHS*, *ELIP1*, and *HYH* was similar to that observed in the *elf3-1* mutants, indicating that the EC could be responsible for the gated response of these genes (Fig. 3B, C; Supplementary S5B,C). Stronger differences in UV-B-dependent induction with respect to the wild type were observed during the subjective night at the time when protein levels of EC components peak (Nusinow *et al.*, 2011). However, a strong constitutive expression of *HY5* was not observed in *elf4-300* or *lux-4* mutants (Fig. 3B, C). These results suggest that ELF3 may play an additional role in UV-B signalling independent of its function within the EC.

### The release of repression observed in *elf3* and *lux4* mutants depends on COP1

**Fig. 4.**
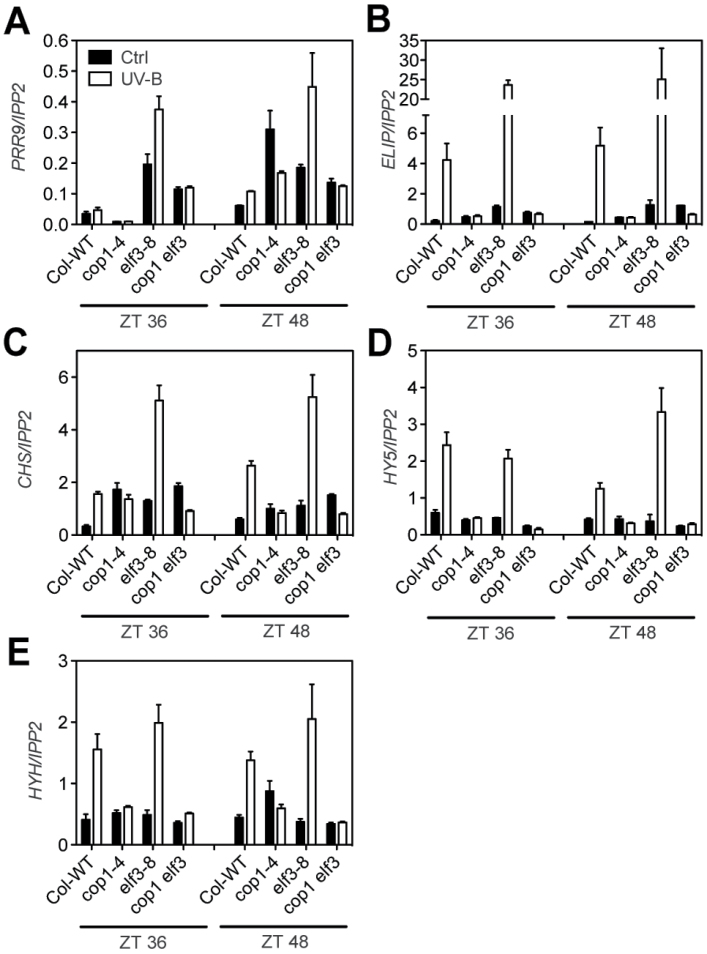
*COP1* functions upstream of *ELF3* on UV-B signalling. RNA levels of *PRR9*, *ELIP1*, *CHS*, *HY5*, and *HYH* in the Col-0 wild type, and *cop1-4*, *elf3-8*, and *cop1-4 elf3-8* mutants under constant light conditions. Two-week-old seedlings were treated with UV-B for 10min at the indicated times using the 345nm (Ctrl) or the 305nm (UV-B) longpass filter. Samples were harvested 1.5h after the start of the treatment. Values represent the averages and standard errors of three biological replicates. The expression levels of each gene were analysed by RT-qPCR and normalized to *IPP2*.

The UV-B-sensing photoreceptor, UVR8, interacts with COP1 to mediate UV-B signals (Cloix *et al.*, 2012). COP1 also regulates the stability of ELF3 protein, and *cop1-4* mutants display elevated levels of ELF3 (Yu *et al.*, 2008). To investigate the role of ELF3 in the expression of UV-B-regulated genes, UV-B-mediated expression was analysed in *cop1-4 elf3-8* double mutants in the subjective morning and subjective night (Fig. 4). As expected, UV-B light did not induce the expression of *PRR9*, *CHS*, *HY5*, *HYH*, and *ELIP1* in *cop1-4*. Moreover, *cop1-4 elf3-8* double mutants had a similar expression pattern to the *cop1-4* mutant. Similar results were observed for *cop1-4 lux-4* double mutants (Supplementary Fig. S6 at *JXB* online). These results show that COP1 is required for an initial step in UV-B perception and confirm that ELF3 and LUX function downstream of COP1 in the circadian gating of the UV-B signalling pathway.

### The evening complex directly regulates the expression of *PRR9* and *ELIP1* but not that of other UV-B-induced genes

**Fig. 5.**
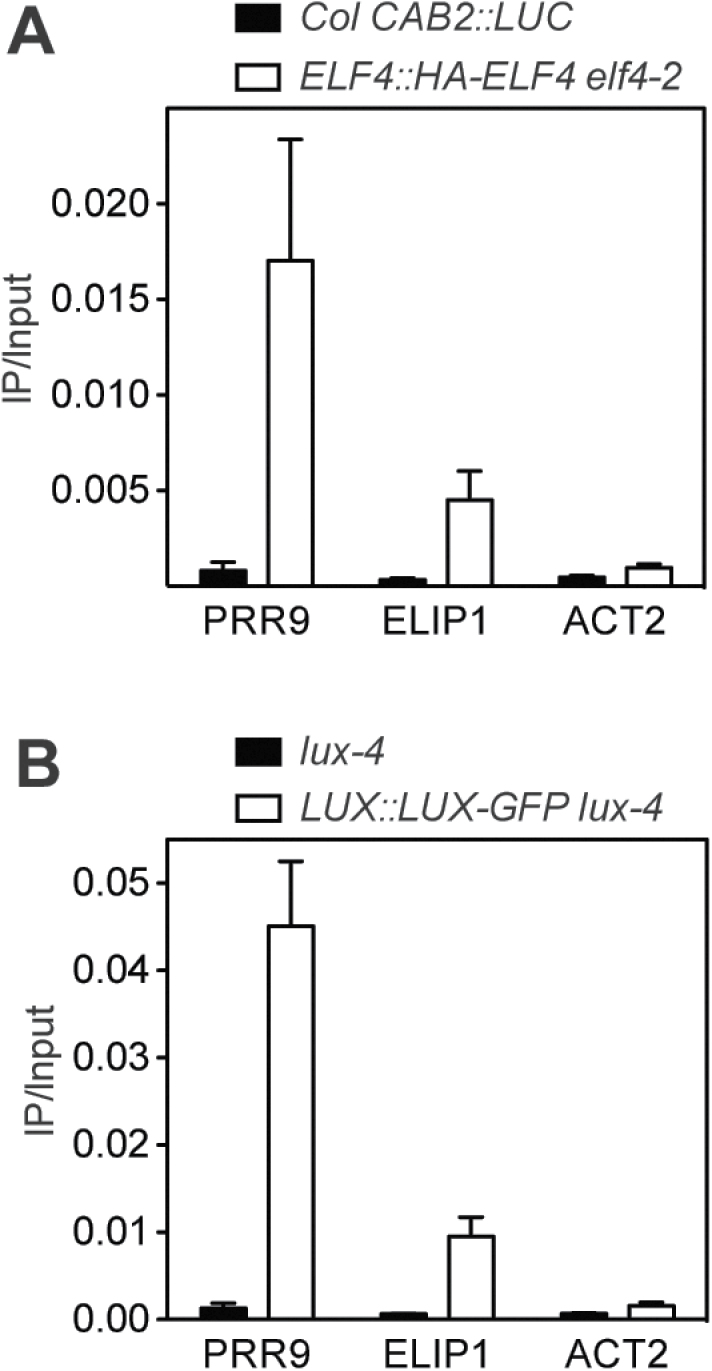
ELF4 and LUX associate with the *PRR9* and *ELIP1* promoters. Chromatin precipitation assays using *ELF4::HA-ELF4 elf4-2* and *LUX::LUX-GFP lux-4* showing the enrichment of promoter fragments co-immunoprecipitated with anti-HA or anti-GFP antibodies, respectively, relative to the input DNA. The enrichment of immunoprecipitated *PRR9* and *ELIP1* promoter regions was analysed by qPCR. Values represent the averages and standard errors of 4–6 independent experiments.

The EC regulates the expression of *PRR9* directly (Dixon *et al.*, 2011; Helfer *et al.*, 2011; Chow *et al.*, 2012; Herrero *et al.*, 2012). Given this direct regulation and the release of gating observed in mutants lacking EC components, it was hypothesized that at least part of the gating response by the clock might be directly mediated by ELF3–ELF4–LUX. The association of ELF4 and LUX with several regions of the *CHS*, *HY5*, *HYH*, and *ELIP1* promoters was investigated by ChIP-qPCR. Lines expressing HA-ELF4 and LUX–GFP under the control of their respective promoters were used (Helfer *et al.*, 2011; Nusinow *et al.*, 2011). ELF4 was associated with the *PRR9* promoter as has been previously reported for LUX and ELF3 (Dixon *et al.*, 2011; Helfer *et al.*, 2011; Chow *et al.*, 2012; Herrero *et al.*, 2012) (Fig. 5). ELF4 and LUX were also found associated with the *ELIP1* promoter (Fig. 5). However, no significant enrichment of ELF4 or LUX was observed in the promoters of *CHS*, *HY5*, and *HYH* (Supplementary Figs S7, S8 at *JXB* online). No effect of UV-B treatment on ELF4 association with chromatin was observed (Supplementary Fig. S7). Taken together, these results suggest that the regulation of the UV-B-induced expression of some genes is caused by direct transcriptional repression by EC components.

### The circadian clock modulates sensitivity to UV-B stress during the night

**Fig. 6.**
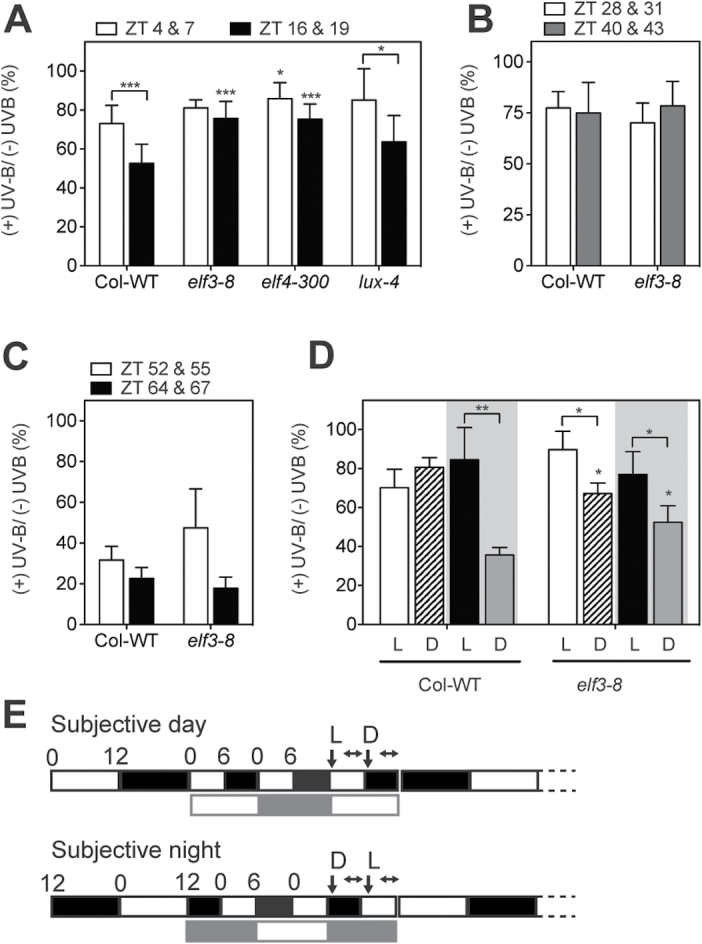
Light and the circadian clock influence plant sensitivity to UV-B stress. For the UV-B treatment (+UVB), 10-day-old seedlings were treated with UV-B using the 305nm longpass filter for 10min at the indicated times (110 μW cm^–2^/3 μmol m^–2^ s^–1^ UV-B). After 3h, the seedlings were irradiated with higher intensity UV-B light for 3h (293 μW cm^–2^/7.7 μmol m^–2^ s^–1^ UV-B). The control seedlings (–UVB) were treated in the same manner but using the 345nm longpass filter. Seedlings were transferred to conditions of 12h light/12h darkness after the treatments. Data represent the ratio as a percentage of the seedling weight between UV-B-treated and control seedlings. The seedlings were weighed 20 d after treatment. The values are the average of 3–14 independent experiments and the standard error of the mean, with the exception of (C) in which *n*=2 independent experiments and the error bars represent the range. In (A–C), the times indicate the time of the pre-treatment and the time of the stress treatment. (A) Seedlings were grown and treated under 12h light/12h dark conditions. (B) Seedlings were transferred to constant light for the times indicated before treatment. (C) Seedlings were transferred to the dark at ZT12. (D) Seedlings were grown under 12h light/12h dark conditions before being transferred to the light regime indicated in (E); shaded areas indicate subjective night periods. In (E), the vertical arrows indicate the time of the pre-treatment and the horizontal arrows the time of the stress treatment. *P<0.05; **P<0.01; ***P<0.001; Student’s *t*-test; differences from the respective wild-type treatment.

In wild-type plants, the circadian clock gates the UV-B induction of gene expression under constant light conditions (Fig. 3) (Feher *et al.*, 2011). However, no time-dependent changes in UV-B stress sensitivity were initially observed (Feher *et al.*, 2011). Moreover, in spite of displaying constitutive UV-B-mediated gene induction, *elf3-*4 mutants appeared to be equally susceptible to UV-B stress as the wild type (Feher *et al.*, 2011). In these experiments, strong UV-B pulses were given to plants grown under constant weak UV-B light. It was investigated whether the combination of a short UV-B pulse followed by a higher intensity UV-B stress revealed changes in UV-B sensitivity at different times of the day. Plant growth was then assessed after a recovery period of 20 d. It was observed that under diel conditions, wild-type seedlings were more sensitive to UV-B stress during the night than during the day (Fig. 6A). This diel difference in sensitivity was absent in the *elf3-8* and *elf4-300* mutants and was weaker in *lux-4* (Fig. 6A). Thus, the lines *elf3-8* and *elf4-300* did not show the increase in UV-B stress sensitivity during the night. To test whether these time-specific changes in sensitivity to UV-B stress were still present under constant conditions, the plants were treated in the subjective day and the subjective night under either constant light or constant darkness. In this case, an overall reduced sensitivity in constant light and increased sensitivity in constant darkness was observed for both the wild-type and *elf3-8* mutant plants (Fig. 6B, C). These results show that visible light is necessary for protection against UV-B light.

In order to investigate further the role of the circadian clock in sensitivity to UV-B stress, the plants were transferred to T-cycles of 6h light and 6h darkness. Wild-type plants cannot entrain to these short cycles and maintain an ~24h period, keeping track of the subjective day and subjective night phases (Kolmos *et al.*, 2011). The circadian clock mediates this phenomenon of frequency de-multiplication. In contrast, *elf3* loss-of-function mutants become arrhythmic under these conditions (Kolmos *et al.*, 2011). The plants were therefore treated with UV-B during the subjective day or subjective night period, either during the 6h light or during the 6h dark phases (Fig. 6D, E). It was observed that wild-type plants were more UV-B resistant during the subjective day regardless of the presence of light (Fig. 6D). However, wild-type plants treated during the subjective night were sensitive to UV-B in the dark but not in the light (Fig. 6D). These results suggest that the circadian clock is able to confer UV-B resistance during the subjective day but light is necessary for resistance during the subjective night. The *elf3-8* mutant was more sensitive to UV-B under T-cycles when treatments were performed in the dark during the subjective night, although they were more resistant than the wild type (Fig. 6D). However, *elf3-8* plants, in contrast to the wild type, were also more sensitive when the UV-B treatment occurred in the dark than in the light during the subjective day. Taken together, these results suggest that the circadian clock is necessary for mediating the sensitivity of plants to UV-B at different times of day.

### Circadian gating of UV-B-induced gene expression also occurs in the dark

**Fig. 7.**
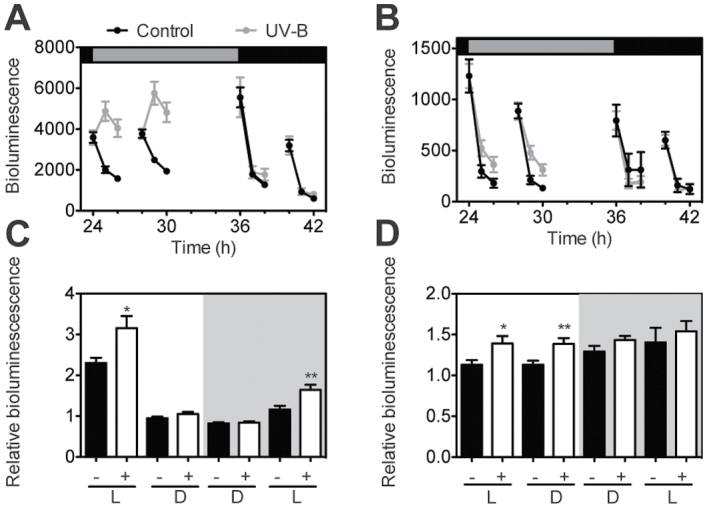
Transcriptional activity of *PRR9pro::LUC*- and *CHSpro::LUC-*expressing lines upon exposure to UV-B irradiation. Plants were treated with UV-B using the 345nm (control) or the 305nm (UV-B) longpass filters. *PRR9pro::LUC* (A, C) and *CHSpro::LUC* (B, D) seedlings were grown for 8 d under 12h light:12h dark before analysis. (A, B) Seedlings were transferred to constant darkness at ZT12 and UV-B treated for 10min; grey areas represent the subjective day and dark areas the subjective night. (C, D) Seedlings were treated with UV-B for 10min at the times indicated by the vertical arrows in Fig. 6E; shaded areas indicate subjective night; (+) indicates UV-B treated seedlings and (–) control seedlings. The data are the average and standard error of 8–16 seedlings. In C and D, *P<0.05 and **P<0.01; Student’s *t*-test, with respect to the control.

It was observed that in wild-type seedlings, light and the circadian clock modulate UV-B stress sensitivity during a diel cycle. It was therefore investigated how light affected UV-B-induced gene expression at different times. Promoter reporter lines of two UV-B-induced genes, *PRR9pro:LUC* and *CHSpro:LUC*, were used (Supplementary Fig. S3 at *JXB* online). It was first observed that the gating of UV-B signals also persisted under constant dark conditions, such that UV-B acted positively on *PRR9pro*- and *CHSpro*-mediated gene expression during the subjective day but not during the subjective night (Fig. 7A, B). UV-B-mediated gene induction was then investigated under T-cycles of 6h light and 6h darkness. Both reporter constructs were induced in the light during the subjective day but not in the dark during the subjective darkness (Fig. 7C, D). However, although *PRR9* expression needed light for UV-B-mediated gene induction under these conditions, *CHS* expression was induced during the subjective day independently of the presence of visible light after UV-B treatment. These experiments show that light and the circadian clock modulate UV-B-mediated gene expression. However, they did not explain the differences in UV-B stress sensitivity observed during the subjective night under T-cycles (Fig. 6D).

## Discussion

The present results show that clock mutants with significantly disturbed circadian rhythms lead to loss of gating of UV-B-mediated gene induction. All the mutants investigated retained UV-B induction of most genes tested. However, it was also observed that circadian clock components that act as transcriptional repressors can inhibit UV-B-induced expression of specific genes. For example, the expression of *ELF4* is strongly repressed in CCA1ox lines even after UV-B treatment (Fig. 2). Under visible light, *ELF4* expression is directly activated by FHR, FAR, HY5, and HYH (Li *et al.*, 2011). The clock components CCA1 and LHY inhibit the positive activity of these proteins by binding to the evening element present in the *ELF4* promoter (Li *et al.*, 2011). It is possible that CCA1 and LHY repress UV-B-mediated induction of *ELF4* expression in a similar manner. Moreover, *PRR7* overexpression inhibits *PRR9* transcription even in the presence of UV-B (Fig. 1A). PRR7 associates with the *PRR9* promoter and also binds to the Groucho/Tup1 co-repressor family, TOPLESS/TOPLESS-RELATED (Wang *et al.*, 2013). Transcriptional repression is likely to be mediated via the TPL association with histone deacetylases (Wang *et al.*, 2013). Interestingly, the expression of *PRR9* is not dependent on HY5/HYH (Feher *et al.*, 2011), and these histone modifications could inhibit transcription activation via a different UVR8–COP1-dependent pathway. Transcriptional control on a gene-by-gene basis could explain how some genes are more UV-B responsive in the morning (*ELIP1*, *CHS*, and *PRR9*) and some in the evening (*ELF4*).

Circadian clock components could also affect UV-B light sensing. Interestingly, although the induction of *HY5* expression by UV-B does not appear to be under circadian control, it is affected in *elf3* loss-of-function mutants and CCA1ox lines (Figs 1, 3) (Feher *et al.*, 2011). Moreover, although the expression of many genes analysed after UV-B treatment was elevated in *elf3*, *elf4*, and *lux* mutants (Fig. 3; Supplementary S5 at *JXB* online), EC components were only found associated with the *ELIP1* promoter in addition to the *PRR9* promoter (Fig. 5), indicating that either other circadian-regulated transcription factors or a transcription-independent mechanism is responsible for these effects. It has been shown that ELF3 binds to COP1 and modulates GIGANTEA (GI) protein levels affecting flowering time (Yu *et al.*, 2008). It is possible that ELF3 could also affect the association of COP1 and UVR8 and modulate UV-B sensing during the night at the peak of ELF3 protein levels (Nusinow *et al.*, 2011). In a similar manner, GI could also affect UV-B signalling by COP1.

The experiments conducted in this study indicate that light and the circadian clock affect the sensitivity of plants to UV-B stress. Plants were more resistant to UV-B light under constant light than under constant dark conditions. This is likely to be due to the inhibition of protective pigment biosynthesis in the dark (Chalker-Scott, 1999). No differences in UV-B stress sensitivity were observed between the subjective day and the subjective night under constant light, although the expression of most UV-B-regulated genes peaks in the middle/end of the night (Supplementary Fig. S2B at *JXB* online). In addition, the *elf3-8* loss-of-function mutants did not have increased resistance to UV-B light (Fig. 6B), in spite of displaying strong and constitutive UV-B-mediated gene induction under constant light conditions (Fig. 4). However, it was observed that wild-type plants under diel cycles were more sensitive to UV-B during the night than during the day and that this difference was reduced in *elf3-8* (Fig. 6A). Experiments under short T-cycles showed that in wild-type plants, darkness affected sensitivity to UV-B stress during the subjective night but not during the subjective day (Fig. 6D). Moreover, *elf3-8* plants retained dark stress sensitivity even during the subjective day. Loss of ELF3 activity leads to arrhythmia and loss of gating of environmental signals under short T-cycles (McWatters *et al.*, 2000; Thines and Harmon, 2010). For example, under these conditions, *elf3* loss-of-function mutants are always responsive to temperature changes (Thines and Harmon, 2010) in a similar manner to what was observed for UV-B sensitivity. These results show that sensitivity to UV-B stress is under circadian control in *Arabidopsis*.

## Supplementary data

Supplementary data are available at *JXB* online.

Figure S1. Spectra of the different UV light treatments between 280nm and 500nm.

Figure S2. Circadian regulation of UV-B-responsive genes.

Figure S3. Transcriptional activity of promoter–luciferase*-*expressing lines upon exposure to UV-B irradiation.

Figure S4. The expression of *LUX* and *ELF3* in CCA1ox and *prr579* seedlings.

Figure S5. Evening complex mutants show constitutive response to UV-B irradiation.

Figure S6. *COP1* functions upstream of *LUX* in UV-B signalling.

Figure S7. Test for ELF4 association with the promoters of UV-B-regulated genes.

Figure S8. Test for LUX association with the promoters of UV-B-regulated genes.

Table S1. Primers used in this study.

Supplementary Data
