# Supplementary material for: Light and the circadian clock mediate time-specific changes in sensitivity to UV-B stress under light/dark cycles
Source: J Exp Bot. 2014 Aug 21;65(20):6003–12. doi: 10.1093/jxb/eru339 (PMC4203133; doi:10.1093/jxb/eru339)
Supplement: Supplementary Data [file supp_65_20_6003__index.html]

Light and the circadian clock mediate time-specific changes in sensitivity to UV-B stress under light/dark cycles — Supplementary Data 

# Light and the circadian clock mediate time-specific changes in sensitivity to UV-B stress under light/dark cycles

## Supplementary Data

Data files

**Files in this Data Supplement:**

- Supplementary Data - Supplementary Data
- Supplementary Data - Supplementary Data
