# Supplementary material for: Light and the circadian clock mediate time-specific changes in sensitivity to UV-B stress under light/dark cycles
Source: J Exp Bot. 2014 Aug 21;65(20):6003–12. doi: 10.1093/jxb/eru339 (PMC4203133; doi:10.1093/jxb/eru339)
Supplement: Supplementary Data [file supp_eru339_SupplementalFigures_V2.pdf]

Figure S1

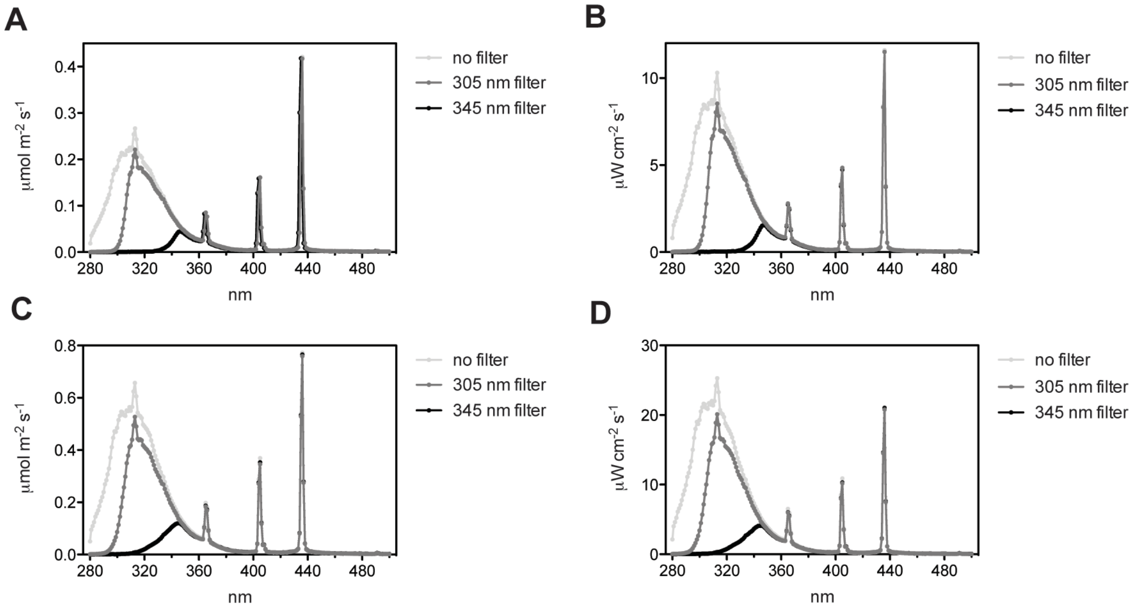

Fig. S1. Spectra of the different UV light treatments between 280 nm and 500 nm. Spectra irradiance was measured in 1 nm intervals under the different long pass filters (305 nm and 345 nm) as well as without filter. (A) and (B) low intensity setting. (C) and (D) high intensity setting. The PS-200 spectroradiometer (Apogee Instruments, US) was used for these measurements.

Figure S2

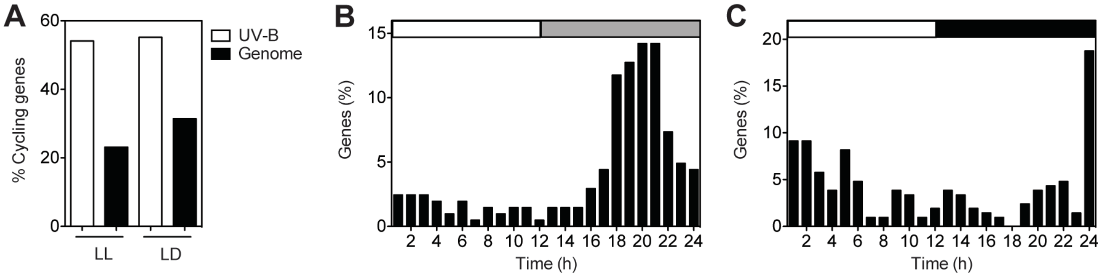

Fig. S2. Circadian regulation of UV-B responsive genes. (A) Percentage of genes that are induced by 1 h UV-B treatment and display cyclic expression in constant light (B) or light/dark cycles (C). UV-B treatment expression data are from (Favory *et al.*, 2005), (2009). Cycling expression data are from (Edwards *et al.*, 2006) for constant light (LL) and from (Blasing *et al.*, 2005) for light/dark (LD). Cycling gene expression was analyzed using PHASER and genes were defined as cycling if model-based pattern matching algorithm >0.8 (Michael *et al.*, 2008).

Figure S3

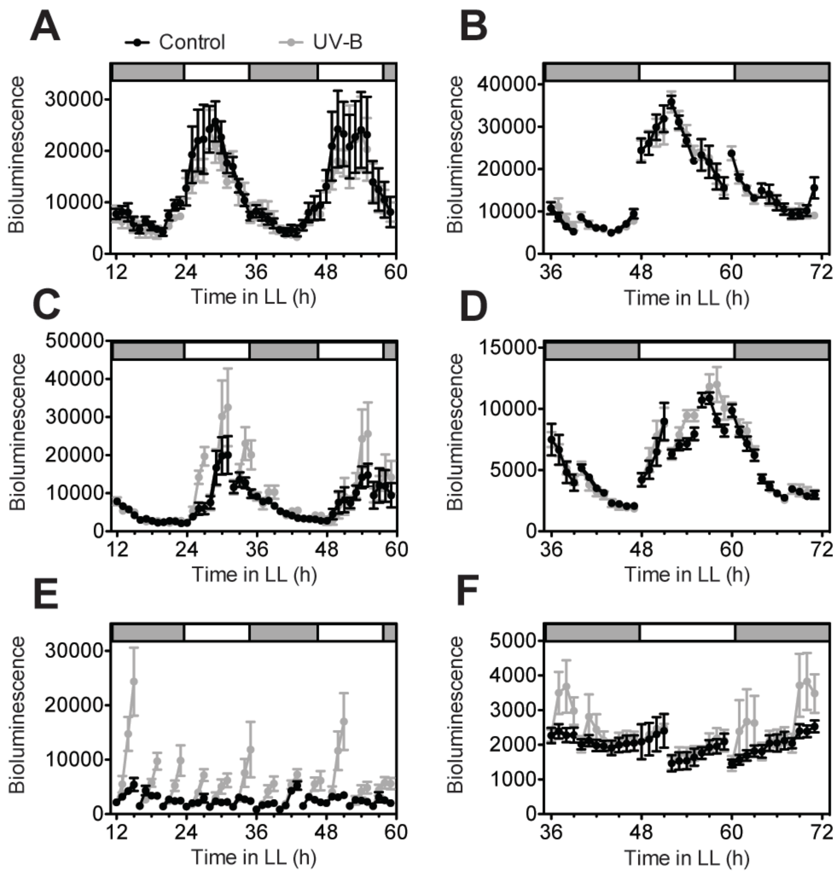

Fig. S3. Transcriptional activity of promoter-luciferase expressing lines upon exposure to UV-B irradiation. Plants were treated with UV-B using the 345 nm (Control) or the 305 nm (UV-B) longpass filters. *CCA1pro::LUC* (A, B), *PRR9pro::LUC* (C, D) and *CHSpro::LUC* (E, F) seedlings were grown for 8 days under 12 h light: 12 h dark before analysis. Seedlings were transferred to constant light conditions at ZT0, and treated with UV-B for 1 h (A, C, E) or 10 min (B, D, F). Shaded areas indicate subjective night. Values are the average and standard error of 6-16 seedlings.

Figure S4

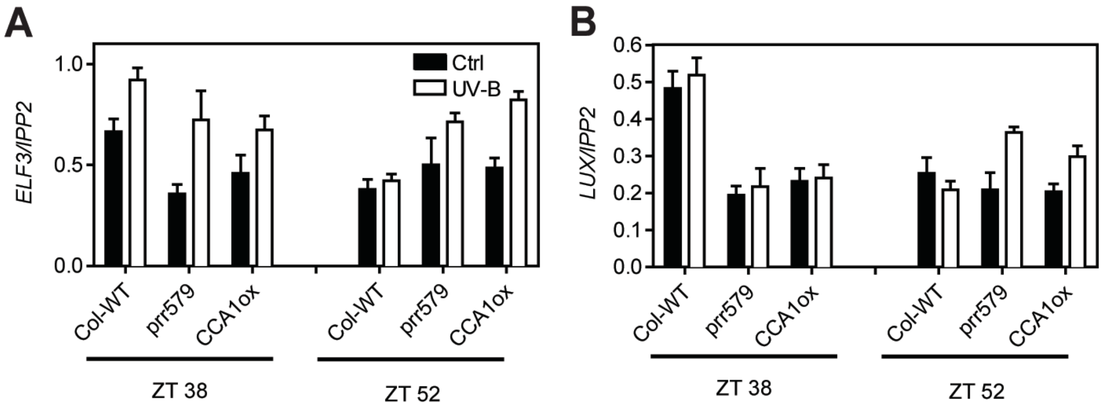

Fig. S4. The expression of *LUX* and *ELF3* in CCA1ox and *prr579* seedlings. Two week-old seedlings were treated with UV-B for 10 min at indicated times under constant light conditions using the 345nm (Ctrl) or the 305 nm (UV-B) long pass filter. Samples were harvested after 1.5 h after the start of the treatment. Values represent the averages and standard errors of three biological replicates. The expression levels of each gene was analyzed by RT-qPCR and normalized to *IPP2*.

Figure S5

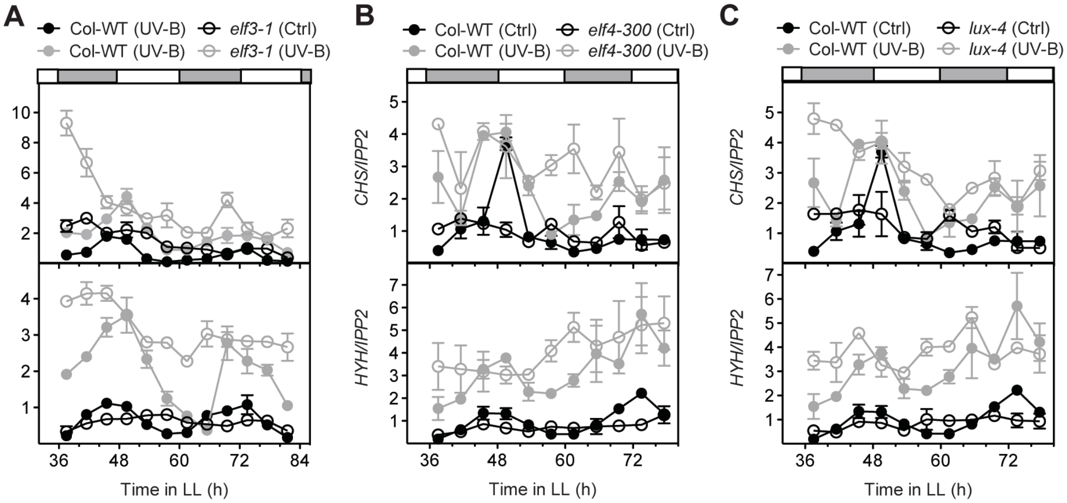

Fig. S5. Evening Complex mutants show constitutive response to UV-B irradiation. Expression levels under constant light conditions in A) *elf3-1*, B) *elf4-300* and C) *lux-4*. Two week-old seedlings were treated with UV-B for 10 min at indicated times using the 345 nm (Ctrl) or the 305 nm (UV-B) longpass filter. Samples were harvested after 1.5 h after the start of the treatment. Values represent the averages and standard errors of three biological replicates. The expression levels of each gene was analyzed by RT-qPCR and normalized to *IPP2*.

Figure S6

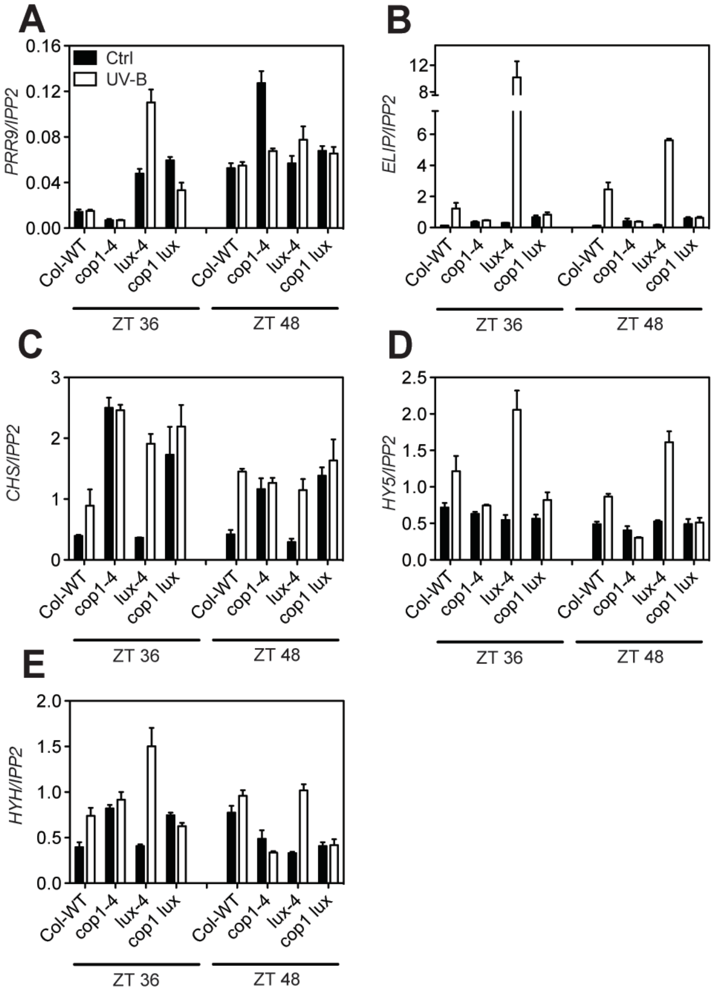

Fig. S6. *COP1* functions upstream of *LUX* in UV-B signaling. RNA levels of *PRR9*, *ELIP1*, *CHS*, *HY5*, and *HYH* in Col-WT and *cop1-4*, *lux-4* and *cop1-4 lux-4* mutants under constant light conditions. Two week-old seedlings were treated with UV-B for 10 min at indicated times using the 345nm (Ctrl) or the 305 nm (UV-B) longpass filter. Samples were harvested after 1.5 h after the start of the treatment. Values are the averages and standard errors of three biological replicates. The expression levels of each gene was analyzed by RT-qPCR and normalized to *IPP2*.

Figure S7

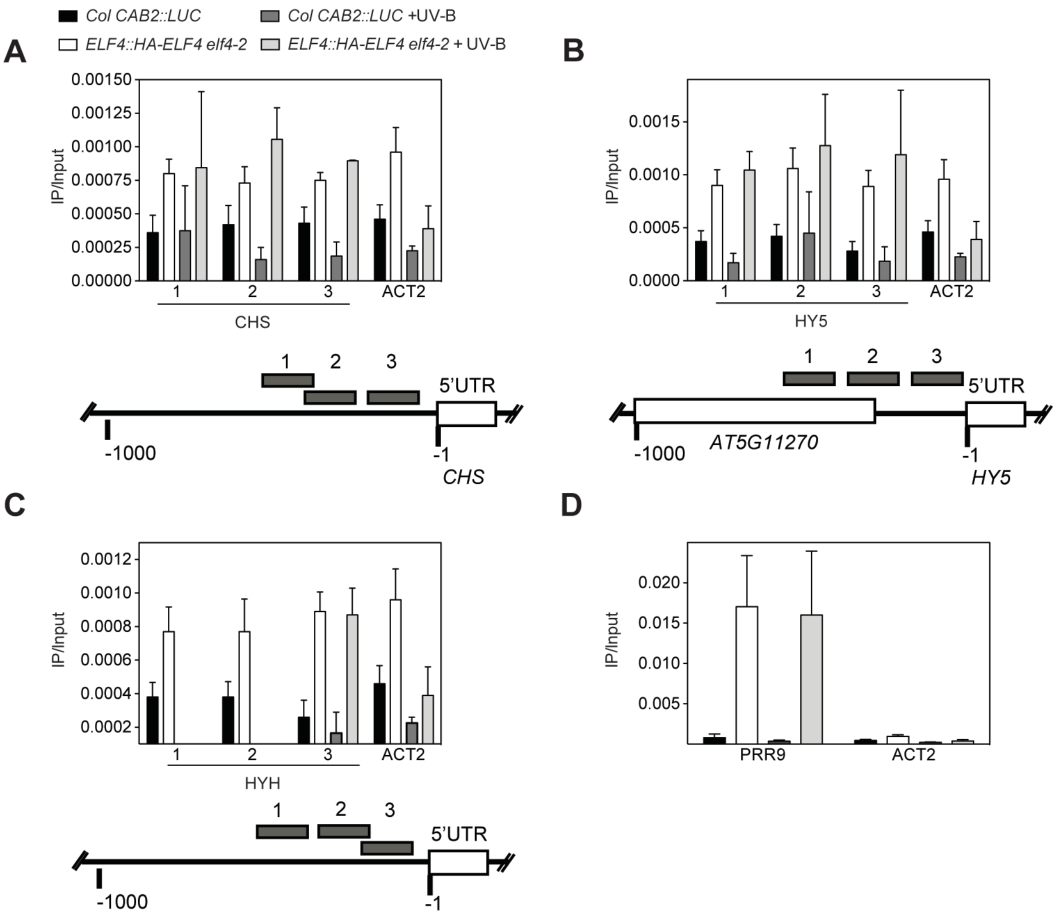

Fig. S7. Test for ELF4 association to the promoters of UV-B regulated genes. Chromatin precipitation assays using *ELF4::HA-ELF4 elf4-2* showing the fraction of DNA fragments co-immunoprecipitated with anti-HA antibodies, relative to the input DNA. For the UV-B treatment, seedlings were transferred to UV-B for 10 min using 305 nm (UV-B) longpass filter 40 min prior to harvest. The presence of promoter regions was analyzed by qPCR, A) *CHS*, B) *HY5*, C) *HYH*, D) *PRR9*. Values are the averages and standard errors of 4 to 6 independent experiments for non-UV-B treated samples and averages and range of 2 independent experiments for the UV-B treated samples. Diagrams indicate the relative positions of the amplified regions.

Figure S8

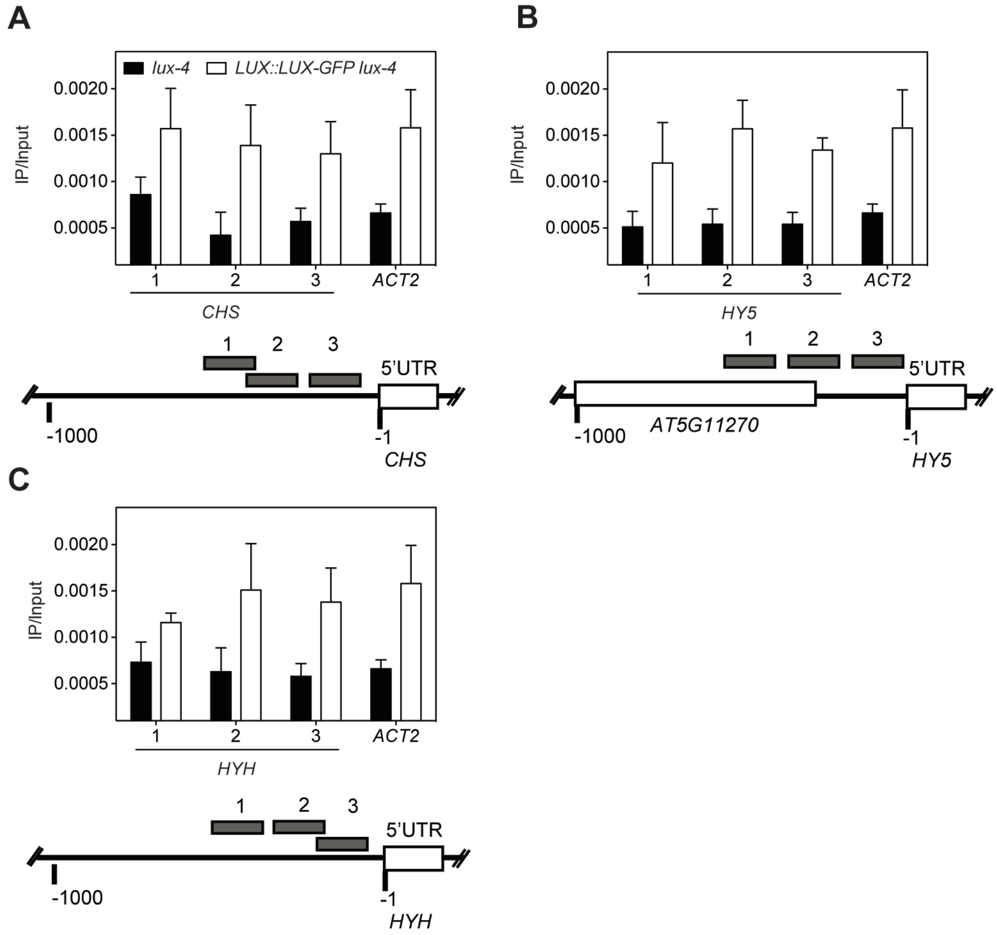

Fig. S8. Test for LUX association to the promoters of UV-B regulated genes. Chromatin precipitation assays using *LUX::LUX-GFP lux-4* showing the fraction of DNA fragments co-immunoprecipitated with anti-GFP antibodies, relative to the input DNA. Promoter regions were detected by qPCR, A) *CHS*, B) *HY5*, C) *HYH*. Values are the averages and standard errors of 4 to 6 independent experiments. Diagrams below indicate the relative positions of the amplified regions.
